# Autophagy-mediated expression clusters are involved in immunity regulation of coronary artery disease

**DOI:** 10.1186/s12863-022-01023-3

**Published:** 2022-04-02

**Authors:** Jin Lv, Dong Wang, Tian Li

**Affiliations:** Cardiovascular Disease Center, The Central Hospital Of Enshi Tujia And Miao Autonomous Prefecture, Enshi, 445000 Hubei China

**Keywords:** Coronary artery disease, Diagnostic classifier, Autophagy, Immunity response

## Abstract

**Background:**

The association between autophagy and immunity, including infiltrating immunocytes, immune reaction gene-sets, and HLAs (human leukocyte antigen) gene, remains unclear. The present study aimed to provide a valid diagnostic tool for coronary artery disease (CAD), and explore the pathological mechanisms of CAD based on the association between autophagy and immunity.

**Methods:**

First, the overlap between differentially expressed genes (DEGs) and autophagy-related genes (ARGs) was identified. Subsequently, machine learning was conducted to screen risk genes closely related to CAD. Diverse autophagy phenotype-related clusters were identified using unsupervised clustering. The connections between different clusters and immune characteristics were evaluated as well.

**Results:**

The present study identified 27 differentially expressed autophagy-related genes (DEAGRs) in CAD samples compared with healthy conrtrols. A classifier constructing by 9 DEARGs was regarded as an effective diagnostic tool for CAD. Furthermore, three distinct autophagy phenotype - related clusters were identified, each cluster exhibited different immune characteristics. Finally, the gene ontology (GO) analysis of 901 autophagy phenotype-related genes showed that immune response, protein phosphorylation, and innate immune response were remarkable enrichment components.

**Conclusions:**

This study identified an effective classifier constituted by 9-DEARGs that has good diagnostic performance for CAD, and revealed that autophagy and the immunity may be common critical factors in the occurrence and development of CAD.

## Introduction

Cardiovascular disease leads to a high incidence and is associated with significant disability and mortality. Annually, 17.7 million people die of cardiovascular disease, accounting for one-third of all deaths globally [1]. By 2030, cardiovascular disease incidence is expected to increase to 23.4 million people globally, accounting for 35% of deaths [2]. Therefore, it is urgent to further study the pathogenesis of CAD and seek accurate diagnostic biomarkers and therapeutic targets for its prevention and treatment.

Current studies have confirmed the complex pathogenesis of CAD; in addition to hyperlipidemia, smoking, obesity, hypertension, diabetes, and other known significant risk factors [3], genetic and external environmental factors are believed to influence CAD development [4, 5]. Autophagy is a major survival mechanism for maintaining cell survival, renewing cellular components, and reusing and stabilizing the internal environment. The main function of autophagy is to remove damaged or aging organelles and maintain a balance of healthy cells [6–8]. The expression levels of autophagy tag genes were found to be abnormal in the pathological process of atherosclerosis. Ox-LDL induces foam cells to reduce autophagosome formation by downregulating LC3II/LC3I and Beclin1 expressions, which is accompanied by P62/SQSTM1 protein accumulation [9]. Ozgur Kaplan et al. [10] observed that ATG5 expression increased in the plasma of patients with coronary artery occlusion. Sergin et al. [11] reported that P62 expression, reflecting autophagy clearance level in human carotid atherosclerotic plaques, increased in areas with heavy plaque load. However, the role of autophagy in CAD pathological process remains largely unknown and requires further exploration. An in-depth investigation of autophagy-related genes (ARGs) in CAD may elucidate potential diagnostic and therapeutic biomarkers. It is widely believed that atherosclerosis is the main cause of CAD. The immune cells in early atherosclerosis accelerate disease progression by interacting with their effector molecules [12]. CAD primarily manifests as an inflammatory disease in which immune mechanisms interact with metabolic risk factors to initiate, propagate, and activate lesions in the arterial tree. Therefore, abnormal inflammatory activation and immune response can induce CAD [13].

Our present study, for the first time, comprehensively explored differentially expressed autophagy-related genes (DEARGs) from the GSE20686 dataset. Further, an autophagy phenotype - related diagnostic model for CAD was established based on the DEARGs using machine learning. Unsupervised clustering was applied to identify autophagy phenotype - related clusters in CAD samples, of which three exhibited diverse immune characteristics. Our investigation discovered that autophagy regulated pathological process of CAD by interacting with immunity mechanisms.

## Materials and methods

### Data acquisition and processing

The GSE20686 dataset contained 99 healthy controls (24 females and 75 males) and 99 CAD samples (24 females and 75 males) was obtained from the GEO database (https://www.ncbi.nlm.nih.gov/geo/query/acc.cgi?acc= GSE20686). All these samples were whole blood. GSE20686 recorded detailed information of patients, including disease status, gender, date of birth, smoking status, nationality and so on. Gene expression was detected by Agilent-014850 Whole Human Genome Microarray 4x44K G4112F. Gene symbols were annotated to gene probes; probes matching multiple gene symbols and those that did not match any gene symbols were excluded.

We used the bioconductor linear models for microarray data (limma) package in the R software to identify differentially expressed genes (DEGs) between healthy controls and CAD samples, with cut-off criteria set as | LogFC | > 0.1 and *P* < 0.05. The Wilcox test was used to compare the expressions of DEARGs between CAD samples and healthy controls.

Gene ontology (GO) and Kyoto Encyclopedia of Genes and Genomes (KEGG) analysis were conducted by “clusterProfiler” to analyze the possible mechanisms involved in the CAD development.

A total of 794 ARGs were obtained from the database http://www.autophagy.lu/. The overlap of ARGs and DEGs were identified and categorized as DEARGs, yielding 27 results. The autophagy phenotype - related pattern genes and gene modules were identified by WGCNA using the R package “WGCNA.”

### Validation of autophagy-related diagnostic signatures

The 27 DEARGs were input into the STRING database (https://string-db.org/) to develop a protein–protein interaction (PPI) network. Spearman correlation analysis was applied to evaluate the expression association among 27 DEARGs. Univariate logistic regression was used to select the more closely DEARGs related to CAD with the cut-off criteria *P* < 0.05; Subsequently, least absolute shrinkage and selection operator (LASSO) was employed for feature selection and gene number reduction, and multicollinearity problem revolve in regression analysis. Multivariate logistical regression with a backwards method was conducted to construct a classifier for diagnosis of CAD. Finally, receiver operating characteristic (ROC) curve analysis was conducted to evaluate the distinguishing performance of autophagy signature in CAD development.

### Diverse cluster identification based on autophagy gene expression

Based on the expression profiles of 27 DEARGs, we used the unsupervised clustering analysis to identify distinct autophagy specific - related clusters. A consensus clustering algorithm was used to evaluate the clustering number and robustness [14]. To ensure the robustness of the classification, the R package “ConsensusClusterPlus” was adopted to conduct 1000 iterations and apply a consensus clustering algorithm [15]. Principal component analysis (PCA) was conducted to further validate the different autophagy expression clusters.

### Estimation of immunity in CAD development

Single-sample gene set enrichment analysis (ssGSEA) was applied to determine the relative enrichment of infiltrating immunocytes and the activity of immune pathways. The gene sets were used to evaluate immunocyte composition, and data for immune-related pathway activity was obtained from previous study [16]. The Wilcox test was used to compare the expressions of DEARGs, the relative immunocyte fraction, immune pathway activity, and human leukocyte antigen (HLA) gene expression among the autophagy expression clusters.

### Biological enrichment analysis for different autophagy expression clusters

The HALLMARKS and KEGG pathways are two commonly pathway gene sets. First, the expression matrix was transformed into the pathway activation score matrix using the gene set variation analysis (GSVA) algorithm. The gene sets of ‘h.all.v7.0.symbols’ and ‘c2.cp.kegg.v7.0.symbols’ were downloaded from MSigDB database for running GSVA analysis. Subsequently, the “limma” package in R was used to compare the pathway activation scores between different subtypes, and *P* < 0.05 was selected as the cut-off criterion.

### Statistical analysis

The mean ± standard deviation (SD) is used to show continuous variables. The t-test or Mann-Whitney U test was conducted to compare differences in continuous variables when appropriate. The chi-squared test or Fisher’s exact test was performed to compare differences in categorical variables as appropriate. Receiver operating characteristic (ROC) curves was used to evaluate the diagnostic efficiency of the classifier. The statistical analyses were performed using R software (version 3.6.2). *P* < 0.05 was considered statistically significant for statistical tests.

## Result

### Differential expression of autophagy-related genes between healthy controls and CAD samples

**Fig. 1:**
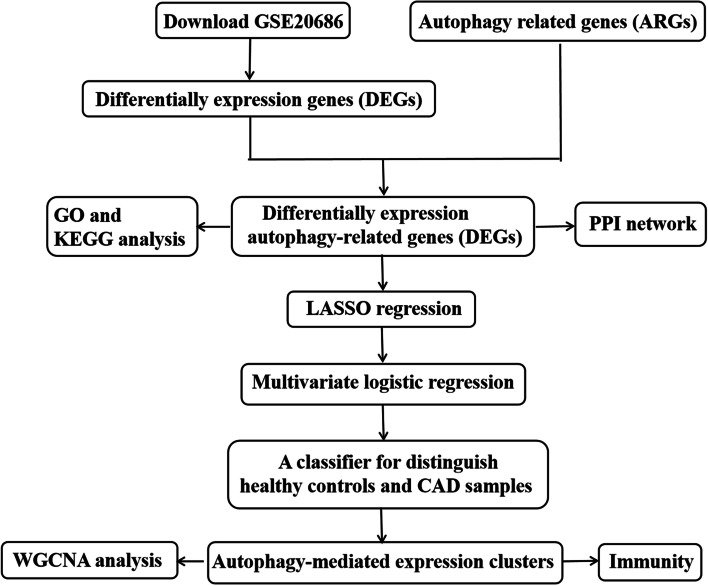
The flow chart of this study

**Fig. 2:**
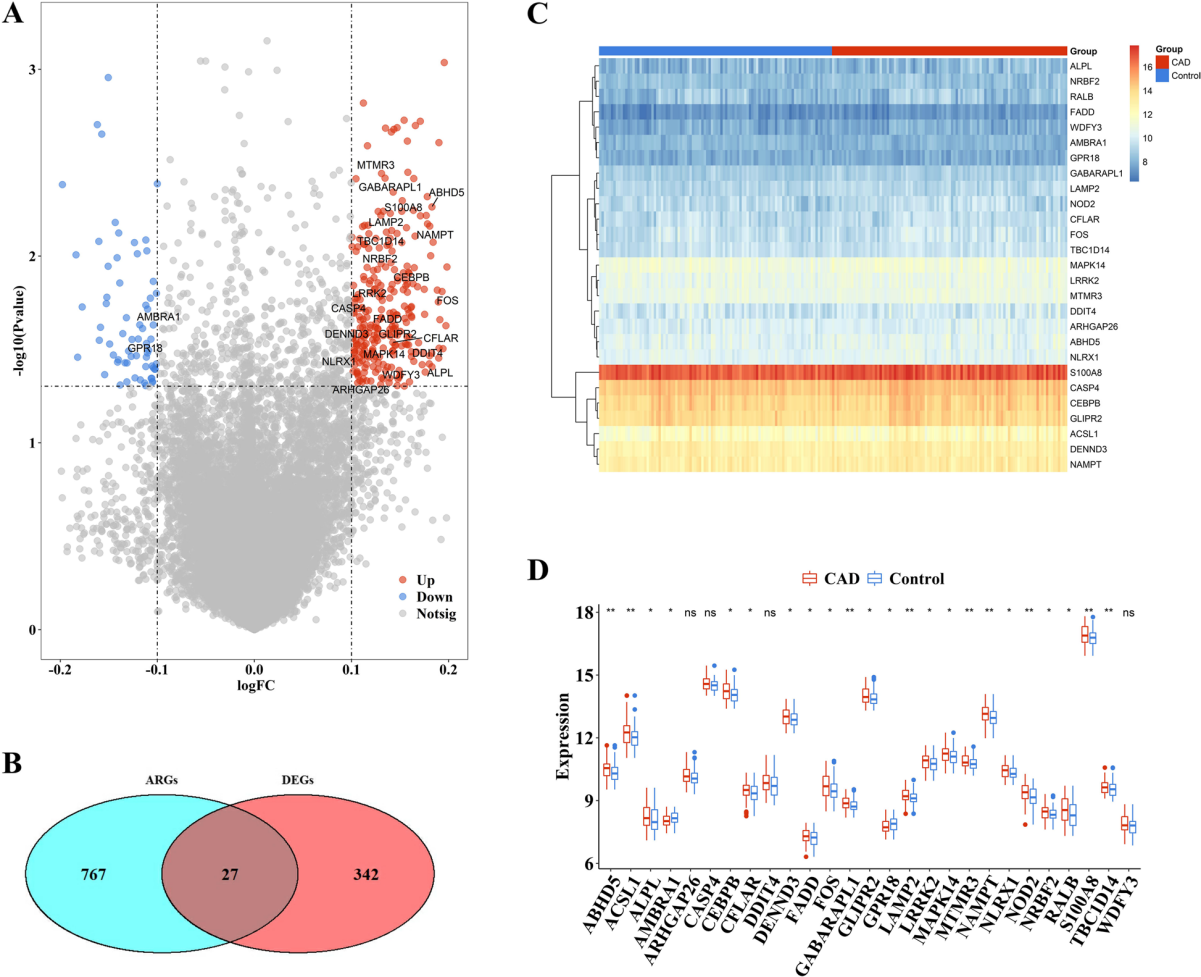
Expression landscape of autophagy genes in CAD. (**A**) The volcano-plot showed the alterations of ARGs between healthy controls and CAD samples. (**B**) The overlap of DEGs and ARGs were DEARGs, totally 27 DEARGs. (**C, D**) The heatmap-plot and box-plot indicated the 27 DEARGs between healthy controls and CAD samples

The flowchart of the present study was illustrated in Fig. 1. First, we extracted expression profile data from the GSE20686 dataset to identify changes in gene expression between CAD samples and healthy controls. In total, compared with healthy controls, 368 DEGs were identified in the CAD group, of which 299 and 69 genes were upregulated and downregulated, respectively (Fig. 2A).

**Fig. 3:**
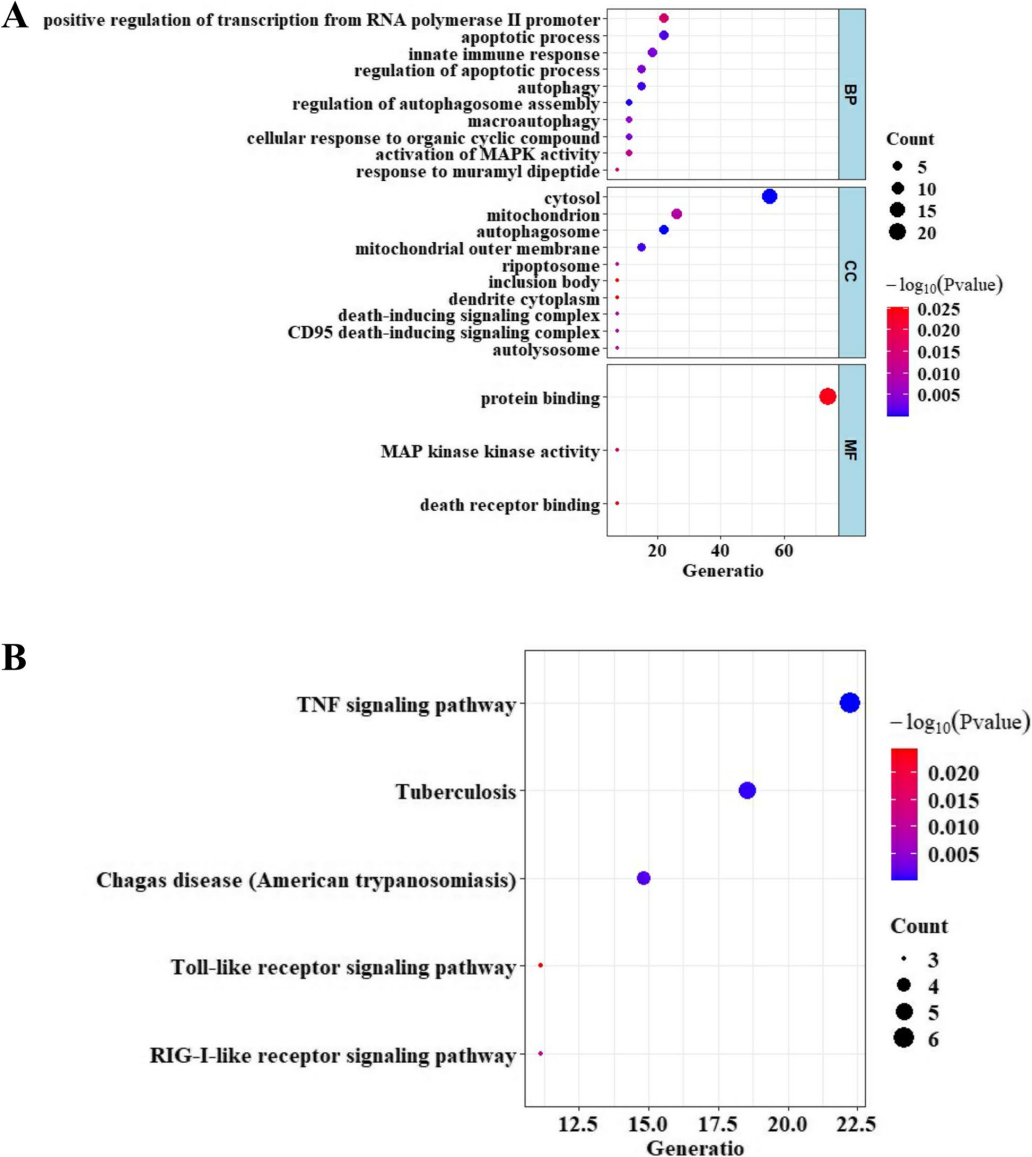
The biological mechanism of 27 DEARGs. (**A**) GO and (**B**) KEGG analysis of 27 DEARGs

To reveal the biological process and mechanisms of DEGs, we conducted GO and KEGG enrichment analyses. The result indicated that the identified DEARGs were associated with 33 biological processes, 16 cell components, and 8 molecular functions. The biological enrichments were mainly related to apoptotic process, innate immune response, and MAPK activity (Fig. 3A). The KEGG analysis revealed that DEGs were mainly enriched in TNF signaling pathway, Tuberculosis, and Toll-like receptor signaling pathway (Fig. 3B).

**Fig. 4:**
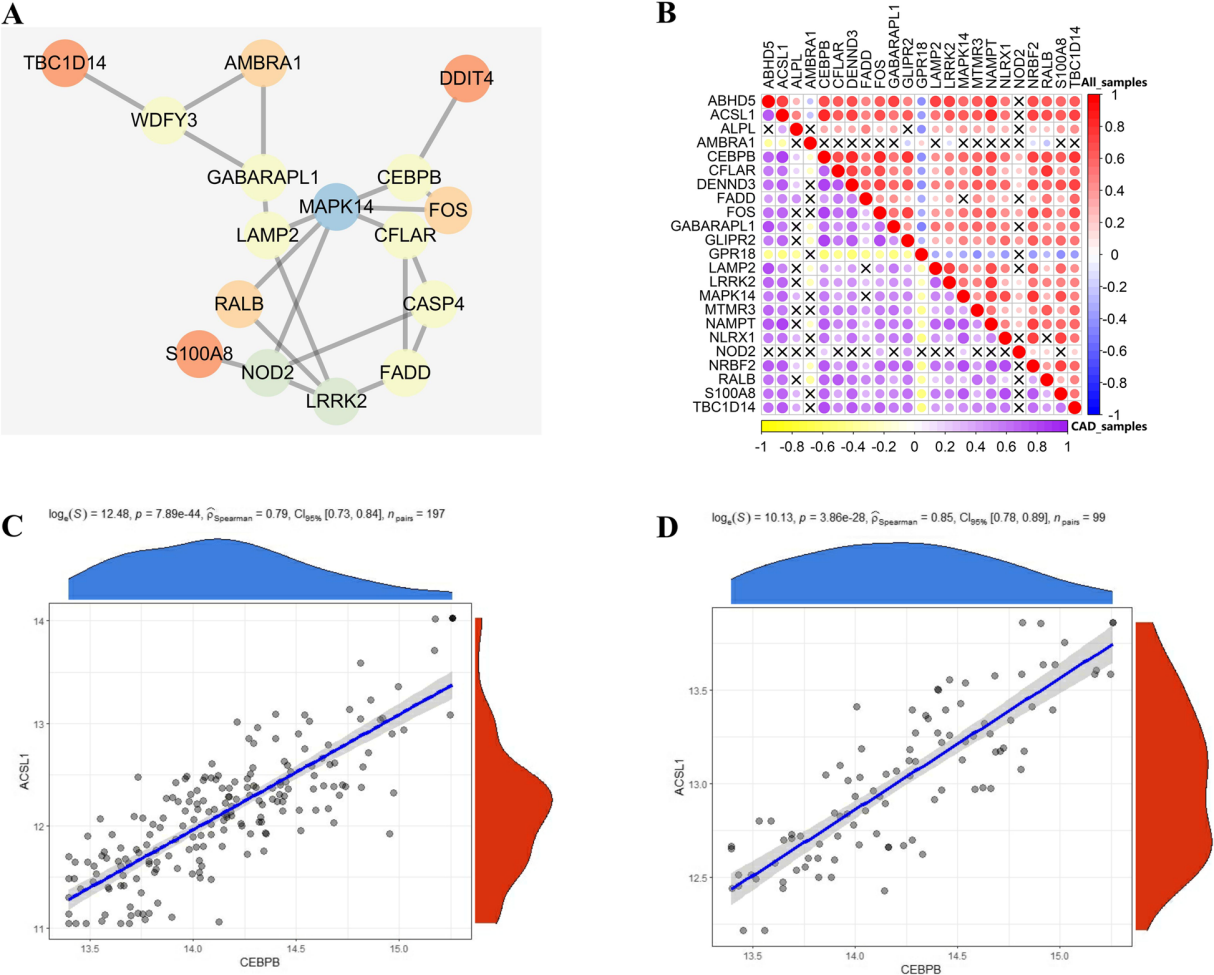
The correlations between DEARGS. (**A**) The protein-protein interactions of 27 DEARGs. (**B**) Correlations among the expression of 27 DEARGs in all samples and CAD samples. (**C, D**) The two scatter-plots demonstrated the most correlated two ARGs: ACSL1 and CEBPB

A total of 794 genes involved in autophagy were acquired. Figure 2B showed that 27 DEARGs were identified by determining the overlapping DEGs and ARGs. Heatmap and boxplot data illustration (Fig. 2C, D) indicated that 22 ARGs exhibited notably higher expression levels in CAD than in healthy samples, while 1 ARGs exhibited the opposite phenomenon. Of the analyzed genes, changes in ARHGAP26, CASP4, DDIT4, and WDFY3 were not statistically significant. PPI network analysis was performed to discover the interaction between the DEARGs (Fig. 4A). The result indicated that ACSL1 and CEBPB were the most highly correlated DEARGs (Fig. 4B, D).

### Construction of the diagnostic classifier for CAD

**Fig. 5:**
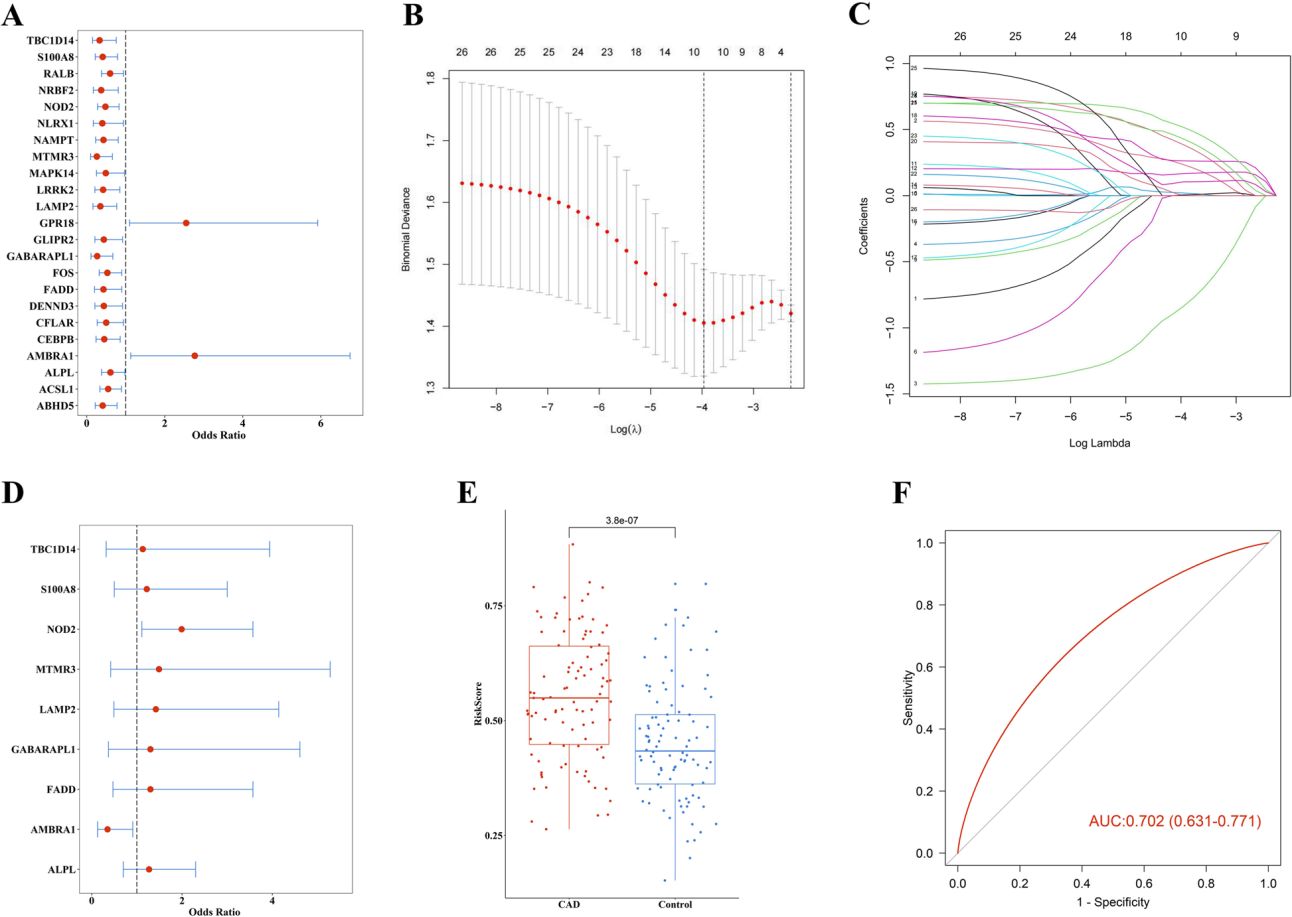
ARGs could distinguish healthy controls and CAD samples. (**A**) Univariate logistic regression of 27 DEARGs. (**B-C**) LASSO regression of DEARGs. (**D**) Multivariate logistic regression of 27 DEARGs, totally 9 DEARGs included. (**E**) The risk score of healthy controls and CAD sample. (**F**) The discrimination ability for healthy controls and CAD samples by DEARGs was analyzed by ROC curve and evaluated by AUC value

We conducted machine learning to construct a diagnostic classifier for CAD according to DEARGs. First, univariate logistic regression was used to screen CAD-related DEARGs, Fig. 5A showed there were 23 genes (*ABHD5, ACSL1, ALPL, AMBRA1, ARHGAP26, CASP4, CEBPB, CFLAR, DDIT4, DENND3, FADD, FOS, GABARAPL1, GLIPR2, GPR18, LAMP2, LRRK2, MAPK14, MTMR3, NAMPT, NLRX1, NOD2, NRBF2, RALB, S100A8, TBC1D14, WDFY3*) selected from 27 DEARGs. Then, LASSO regression was performed for further feature selection, dimension reduction, and unimportant factors elimination. The LASSO model identified 23 genes (Fig. 5B, C). Subsequently, we passed there genes into multivariate logistic regression to construct a diagnostic classifier for distinction CAD samples from healthy controls, 9 DEARGs (*TBC1D14, S100A8, NOD2, MTMR3, LAMP2, GABARAPL1, FADD, AMBRA1, ALPL*) were obtained (Fig. 5D).

The risk score was measured for each sample based on this classifier. The result revealed that CAD samples had a much higher risk score than that of healthy samples (Fig. 5E). The ROC was used to evaluate the diagnostic efficiency of this classifier. Figure 5F showed that the 9 DEARGs comprise a system that may be effective for differentiating healthy controls and CAD samples (AUC = 0.702).

### Identification of diverse autophagy-related clusters

**Fig. 6:**
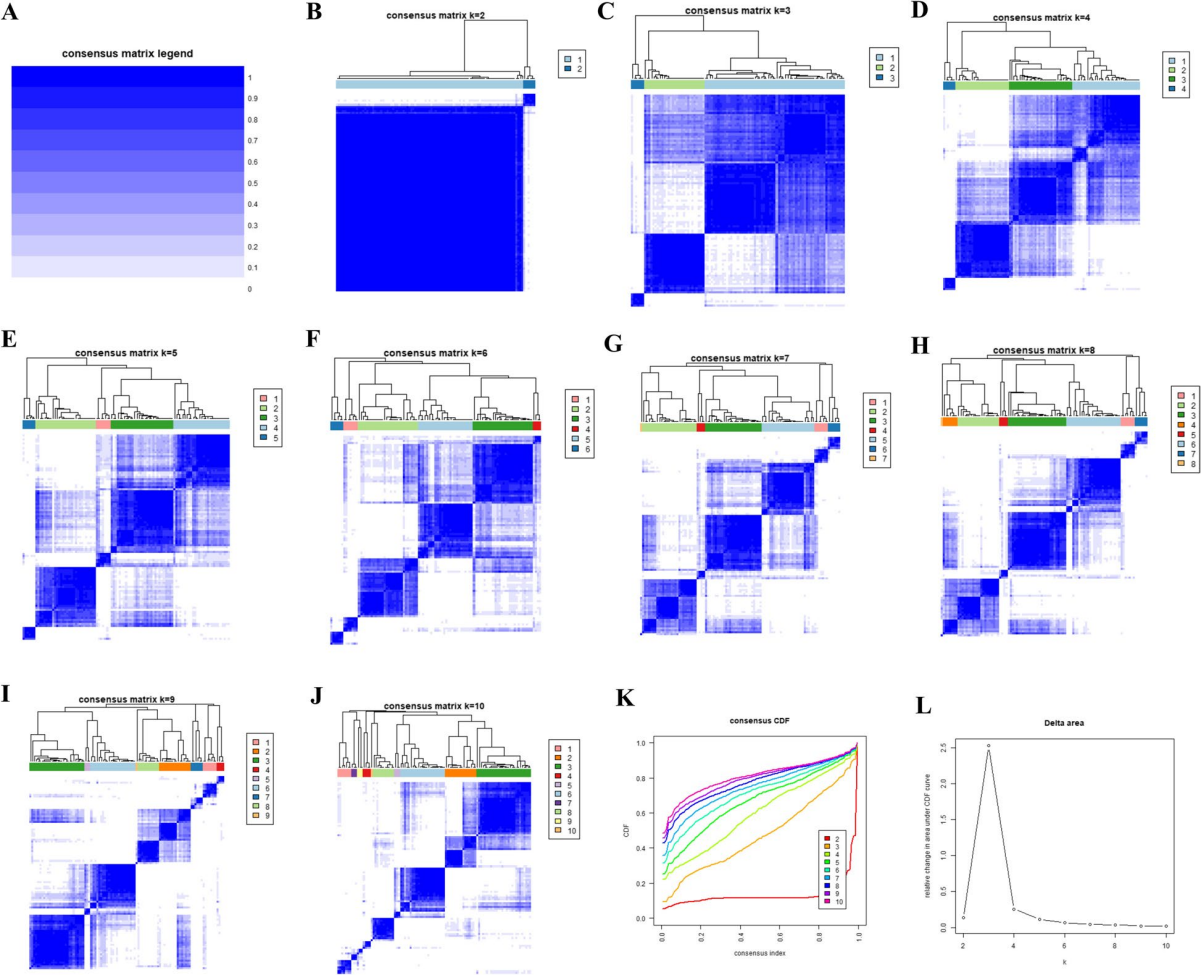
Unsupervised clustering of 27 DEARGs. Identifying 3 diverse autophagy-mediated expression clusters in CAD. (A-J) Heatmap of the matrix of co-occurrence proportions for CAD samples. (K) Consensus clustering cumulative distribution function (CDF) for k = 2–10. (L) Relative change in area under CDF curve for k = 2–10

**Fig. 7:**
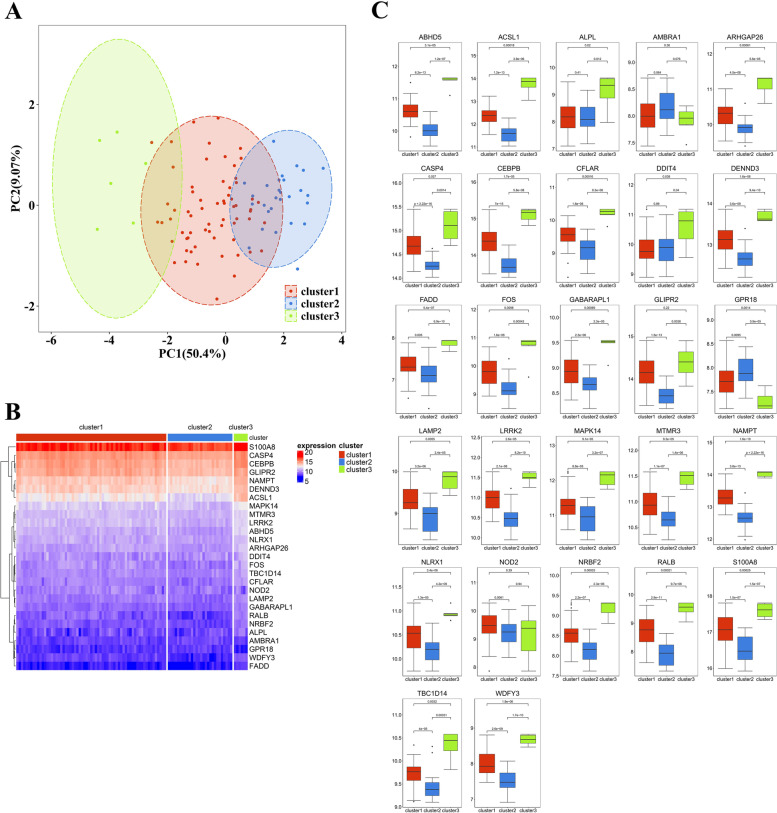
The feature of each autophagy-mediated expression cluster. (A) Principal component analysis (PCA) for the transcriptome profiles of 3 clusters. (B) Unsupervised clustering of 27 DEARGs in the 3 clusters. (C) The expression status of 27 DEARGs in the 3 clusters

Based on the expressions of 27 DEAGRs, we used the unsupervised consensus clustering to classify the CAD patients with different ARGs expressions (Fig. 6). Three clusters of CAD samples were identified: cluster 1 with 65 samples, cluster 2 with 28 samples, and cluster 3 with 6 samples. The result of PCA analysis demonstrated that there was a significant difference in expression profile among these three clusters (Fig. 7A). Comparing the expression of ARGs revealed that, except *GLIPR2* and *NOD2*, The remaining 25 ARGs exhibited remarkable changes in transcriptome profiles among each cluster (Fig. 7B, C), suggesting that autophagy participates in regulation of CAD development.

### Distinct immune characteristics exhibited by autophagy-mediated expression clusters

**Fig. 8:**
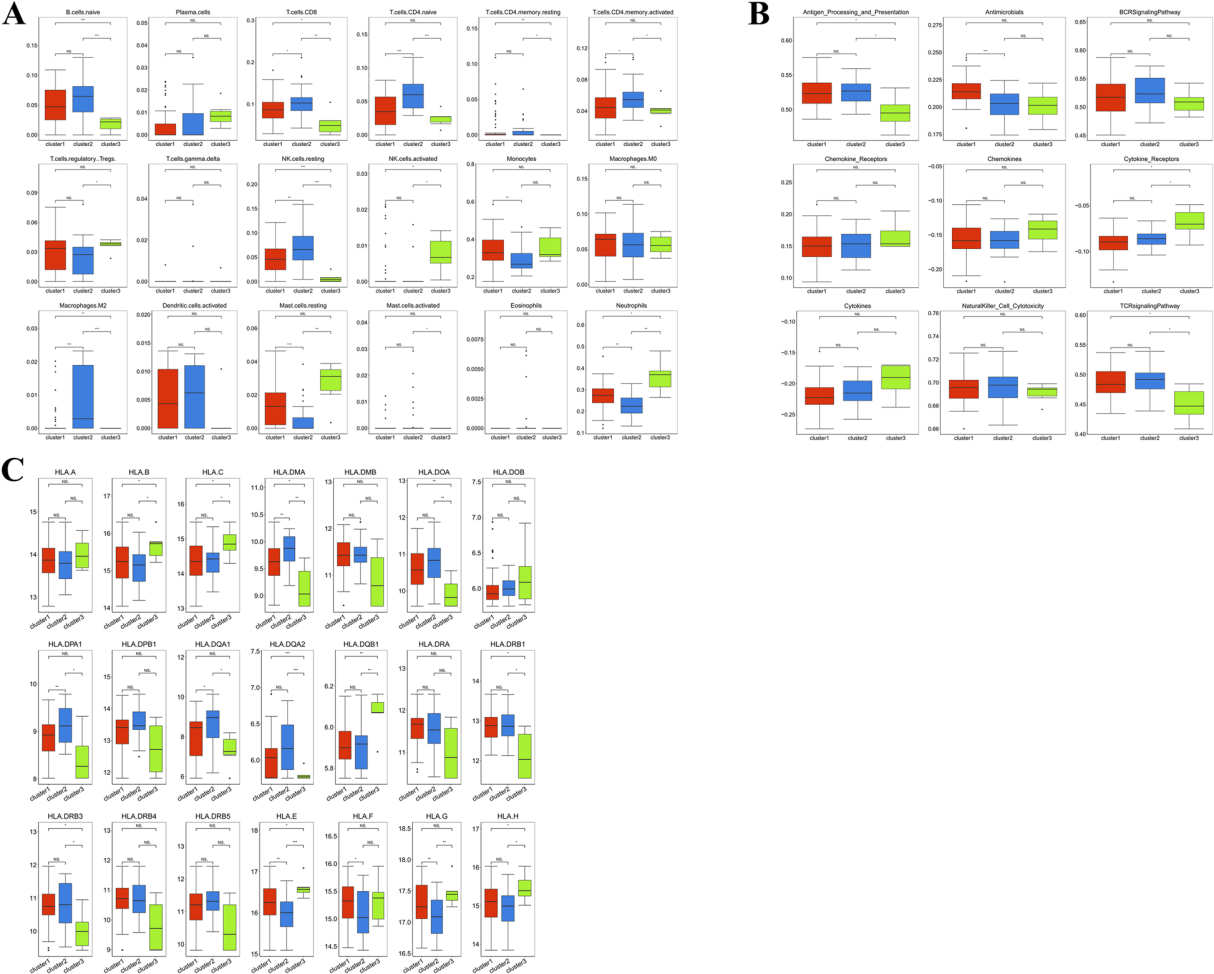
Characteristics of immune microenvironment among autophagy-mediated expression clusters. (**A**) The abundance differences of infiltrating immunocyte among autophagy-mediated expression clusters. (**B**) The activity differences of each immune reaction gene-set among autophagy-mediated expression clusters. (**C**) The expression differences of each HLA gene among autophagy-mediated expression clusters

CAD is an inflammatory status with long-lasting activation of innate immunity. Previous study reported that immune imbalance was present in the CAD patients, and was associated with changes of disease markers, including higher stenosis severity and less calcified lesions [17]. To elucidate the differences in immunity characteristics among these three autophagy-related clusters, we assessed infiltrating immunocytes, immune reaction gene sets, and HLA gene expression. First, many infiltrating immunocytes differed among these three clusters (Fig. 8A). Excluding plasma cells, gamma delta T cells, MO macrophages, activated dendritic cells, and eosinophils, other immunocytes appeared to exhibit changes in abundance among these three clusters. Second, for immune reactions, cluster 3 showed different results compared with clusters 1 and 2. Cluster 3 exhibited a low activity in antigen-processing and presentation, antimicrobial, and TCR signaling pathways; however, clusters 1 and 2 exhibited the opposite trend. Cytokine receptors were more active in cluster 3 than in clusters 1 and 2 (Fig. 8B). Third, HLA gene expression analysis across the three clusters provided results similar to those shown above. Most HLA genes, such as *HLA.DMA, HLA.DOA, HLA.DPA1, HLA.DQA1, HLA.DRB1, HLA.DRB3, HLA.DRB4, and HLA.DRB5*, had significantly lower expressions in cluster 3 than in clusters 1 and 2, indicating that cluster 3 regulated a mild immune response. However, clusters 1 and 2 mediated an active immune response (Fig. 8C). Our result revealed that the autophagy and immunity worked together to regulate the pathological process of CAD.

### Biological characteristics of autophagy-related clusters

**Fig. 9:**
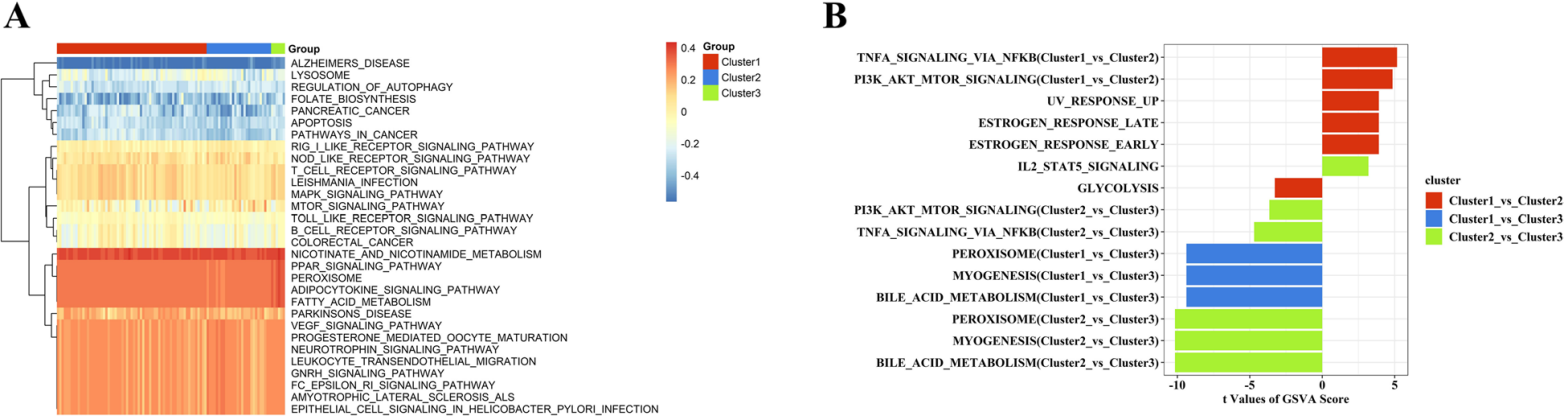
The underlying biological function characteristics diversity among autophagy-mediated expression clusters. (**A**) The HALLMARKS pathway enrichment score among autophagy-mediated expression clusters. (**B**) The KEGG analysis enrichment score among autophagy-mediated expression clusters

**Fig. 10:**
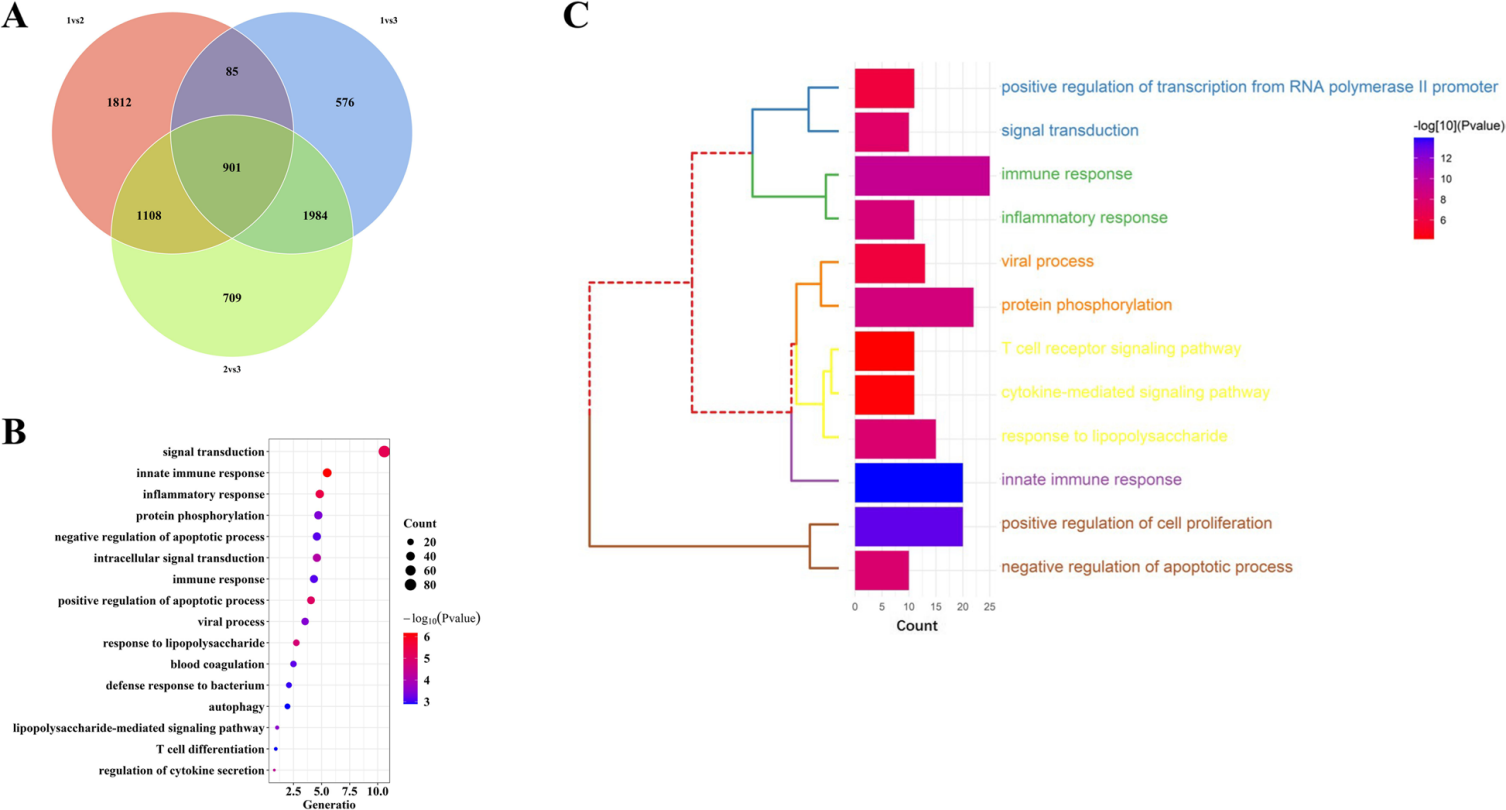
Identification and function analysis of autophagy-related genes in CAD. (**A**) 901 autophagy-related genes were exhibited in Venn diagram. (**B**) The biological characteristics of autophagy-related genes were analyzed by GO-BP. (**C**) The autophagy-related immune genes showed the relationship between autophagy and immune regulations by the GO-BP enrichment analysis

To comprehensively elucidate the molecular functions of these clusters, we conducted GSVA and GO-BP analysis. GSVA analysis was used to measure the enrichment scores of LLMARK and KEGG pathways in the clusters. The results showed that many pathways were enriched in cluster 1, including the TNF-α and PI3K-AKT-mTOR signaling pathways (Fig. 9). Moreover, we selected 901 overlap genes as key genes in the autophagy process; GO enrichment analysis revealed that these genes were mainly involved in innate immune and inflammatory response pathways (Fig. 10A, B). The autophagy phenotype-related genes involved in immune regulation were selected and used for GO-BP analysis. Immune response, protein phosphorylation, innate immune response, and positive regulation of cell proliferation were selected as key determinants of the remarkable enrichment component (Fig. 10C).

### Correlation of comprehensive gene landscape with each autophagy-mediated expression cluster

**Fig. 11:**
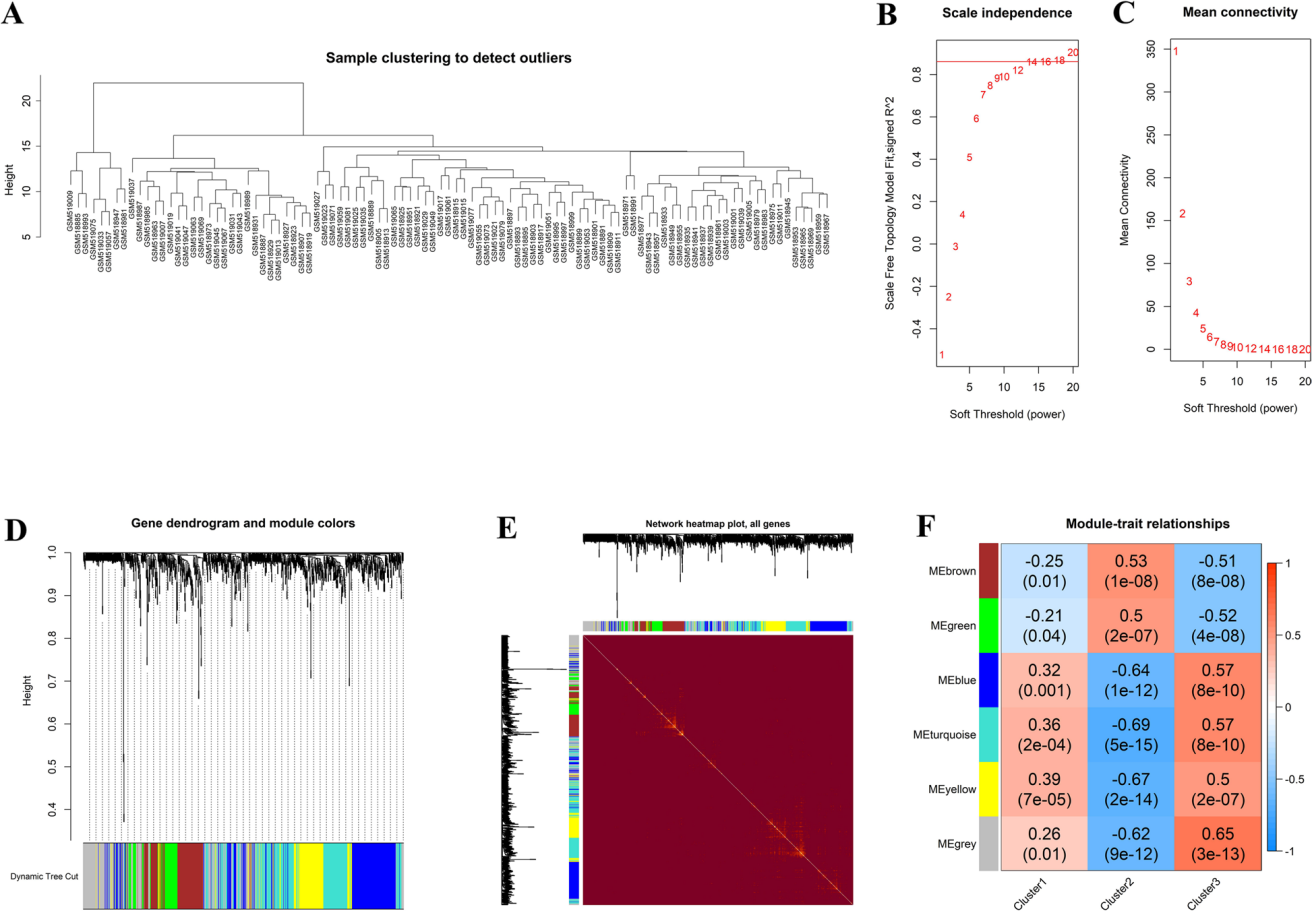
Genes and gene modules related to autophagy-mediated expression clusters. (**A**) The sample clustering was based on the expression data of all samples. (**B-C**) The most appropriate soft threshold was calculated. (**D**) Gene dendrogram obtained by average linkage hierarchical clustering. (**E-F**) Heatmap of the correlation between module eigengenes and the autophagy-mediated expression clusters

**Fig. 12:**
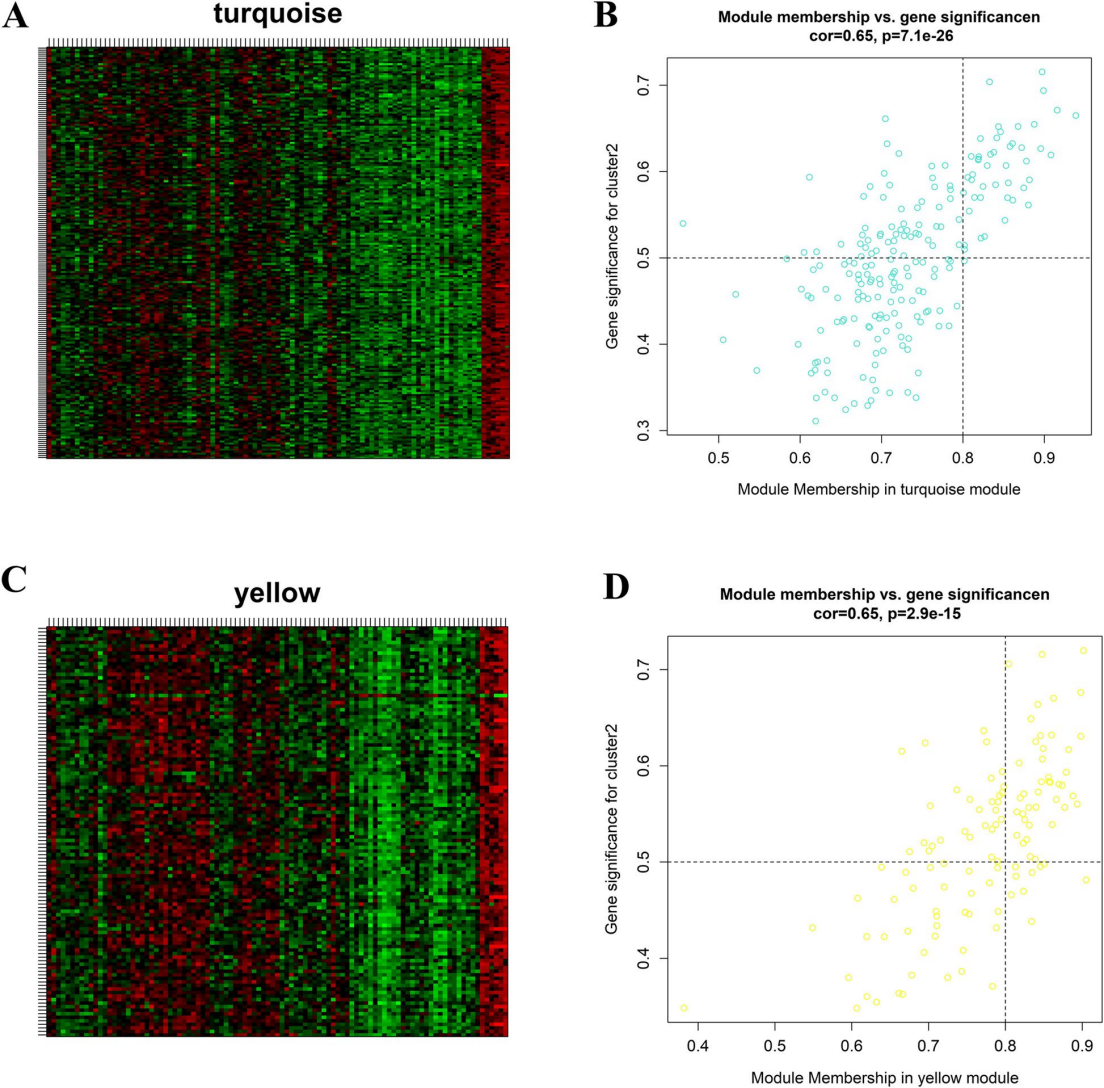
Correlation of different gene modules and clusters. (**A, C**) Heatmap of turquoise and yellow modules gene expression. (**B, D**) A scatterplot of gene significance (GS) for autophagy-mediated expression cluster-2 versus module membership (MM) in the turquoise and yellow modules

After screening the significant DEGs among the three clusters, 901 genes were selected for WGCNA analysis to identify gene–gene modules related to each cluster. In total, six gene modules were identified. There were 196, 118, 65, 200, 204, and 116 genes in the blue, brown, green, grey, turquoise, and yellow modules, respectively (Fig. 11A-11E). Correlation analysis indicated cluster 2 was highly associated with genes in the turquoise (− 0.69, 5e-15) and yellow (− 0.67, 2e-14) modules (Fig. 11F). We extracted the genes from these two modules regarding hub genes according to the cut-off criteria of gene significance > 0.5 and module membership > 0.8. These hub genes tended to be closely correlated with autophagy-mediated expression cluster 2 (Fig. 12A-D).

## Discussion

To the best of our knowledge, this study is the first to systematically investigate the role of ARGs in the diagnosis of CAD, and the association of autophagy and immunity in the pathology of CAD. Firstly, compared with healthy controls, 27 ARGs were identified as having the most remarkable abnormal expressions in CAD samples. Secondly, a classifier, including 9 DEARGs, having good diagnostic performance for CAD.

We found several genes might be highly significant because of their large fold change and significance in regression analysis, especially *S100A8*, *NOD2*, and *LAMP2*, which have been previously reported to participate in the development of CAD. S100A8, located on chromosome 1q21, is an important member of the S100 multigene subfamily of cytoplasmic EF-hand Ca^2+^-binding proteins [18]. In patients with CAD, S100A8 level increased in blood and coronary artery occlusion sites, and could be used to effectively predict the risk of CAD mortality [19, 20]. It has been demonstrated that the S100A8 profile correlated closely with levels of systemic inflammatory markers and the severity of CAD in patients with diabetes [21]. In addition, S100A8 also possessed a prognostic value, because acute coronary syndrome patients with elevated S100A8 levels exhibited a higher risk of adverse cardiovascular events [22]. Nucleotide-binding oligomerization domain 2 (NOD2), an intracellular pattern recognition receptor of the NOD-like receptor family, is involved in propagating the immune response [23]. Opitz et al. has reported that NOD2 can activate nuclear factor κB (NF-κB) in the human fibroblast and aortic endothelial cell lines in response to *Chlamydia pneumoniae*, one of the most common bacterial species examined in atherosclerotic plaques [24]. Moreover, as a major protein of the lysosomal membrane, LAMP-2 functions as a necessary factor in lysosomal fusion with autophagosomes and phagosomes, lysosomal mobility, and chaperone-medicated autophagy [25]. Higher gene expression of LAMP-2 has suggested an increase in lysosomal accumulation and storage in the leukocytes of patients with CAD [26]. Fas-associated death domain-containing protein (FADD) plays a vital role in death receptors (DR) pathway [27]. Functionally, the death effector domain promotes procaspase-8 aggregation within the death-inducing signaling complex and facilitates its autoproteolysis [28]. AMBRA1, an endogenous PP2A-interacting protein, is a important regulator of autophagy in vertebrates. However, there were no reports on the relationships between *TBC1D14*, *MTMR3*, *GABARAPL1*, and *AMBRA1* with CAD. The findings of our study will provide direction for future in-depth investigation based on these ARGs with CAD.

Inflammation and autophagy are both involved in the pathophysiological procedures of various diseases. Inflammation is a protective mechanism by which organisms defend against pathogens or tissue damage. However, excessive inflammatory response may lead to tissue damage and disease. Study has reported that atherosclerosis and plaque stabilization are mediated by immune infiltration and inflammatory factors. The level of circulating inflammatory mediators is significantly associated with the occurrence, progression, and prognosis of CAD [29]. Besides, autophagy protects cells and acts as an anti-inflammatory by removing sterile stimulants or pathogens destroying cells. Many studies have demonstrated that autophagy directly regulates the secretion of inflammatory cytokines by inhibiting the inflammatory response pathway [30]. Nevertheless, no systematic studies have been conducted to illustrate the communicative functions of autophagy and immunity in patients with CAD. To address this gap, we comprehensively evaluated the potential mechanisms of DEARGs and immune characteristics in CAD. Unsupervised clustering of CAD samples based on the DEARGs expression profiles divided all CAD samples into three clusters, and each cluster exhibited unique immune characteristics. Multiple immune-response molecules have been reported to participate in CAD, especially IL-1R, interferon-gamma (IFN-gamma), tumor necrosis factor-alpha (TNF-alpha), and IL-6 receptor (IL-6R) [31]. Furthermore, in acute coronary syndromes, local cytotoxic CD8+ T cell responses may trigger endothelial damage and plaque erosion. Macrophages can help maintain the local inflammatory response, propagate plaque development, and promote thrombosis in each vascular bed. Neutrophils functionally induce the activation of endothelial cells, antigen-presenting cells, and platelets, resulting in a pro-inflammatory immune response [32]. Observing the differences in immune characteristics among the three clusters of CAD samples could help us understand the potential immune-related mechanisms in the CAD so as to provide strategies for immunotherapy interventions for CAD.

This study was the first to systematically analyze the relationship between autophagy and the immunity in CAD. Based on the relationship between autophagy and immunity, the present study opened a new direction for CAD pathogenesis. However, there were several limitations in this study. First, the methodology of our study was only based on the bioinformatics analysis. Further research should be conducted to verify the results presented herein and validate these findings. Second, although the AUC of the classifier exhibited acceptable discriminatory ability, the model performance needs to be improved. Thus, independent studies with larger sample sizes are warranted to validate and improve the clinical utility of this classifier.

There were two previous studies (PMID: 21443790 and PMID: 23210427) based on GSE20686 dataset, however, there were several differences between present study and them. First, the included population were different; Second, the analyses used in our study and original ones were different as well. Third, the classifier found in our study was based on the autophagy genes, furthermore, we explore the potential mechanism according to the association between autophagy and immune.

In all, this study comprehensively evaluated the functions of autophagy in the diagnosis of CAD, we identified a 9-genes (*TBC1D14*, *S100A8*, *NOD2*, *MTMR3*, *LAMP2*, *GABARAPL1*, *FADD*, *AMBRA1*, *ALPL*) classifier for the first time that may represent diagnostic biomarker for CAD. Moreover, we demonstrated that autophagy and the immunity might be critical factors in the occurrence and development of CAD. These findings elucidated a new direction for understanding the pathogenesis of CAD.

## Data Availability

The datasets presented in this study can be found in online repositories (https://www.st-va.ncbi.nlm.nih.gov/geo/query/acc.cgi?acc=GSE20686).
